# The centrosome and cell proliferation

**DOI:** 10.1186/1747-1028-1-26

**Published:** 2006-11-16

**Authors:** Vlastimil Srsen, Andreas Merdes

**Affiliations:** 1 Wellcome Trust Centre for Cell Biology, University of Edinburgh, King's Buildings, Edinburgh EH9 3JR, UK

## Abstract

Centrosomes are frequently amplified in cancer cells. Increased numbers of centrosomes can give rise to multipolar spindles in mitosis, and thereby lead to the formation of aneuploid daughter cells. However, whether centrosome amplification is a cause or a consequence of cancer is unclear. In contrast, loss of a functional centrosome has been shown to lead to cell cycle arrest. In this review, the potential mechanisms underlying centrosome amplification and centrosome-dependent cell cycle regulation are discussed.

## Background

The centrosome is the major microtubule organizing center in proliferating human cells. It is a small organelle composed of two cylindrically shaped centrioles that are surrounded by pericentriolar material. The centrosome duplicates during S-phase: in this process the two centrioles separate and serve as templates for the formation of new daughter centrioles. The duplicated centrosomes accumulate additional pericentriolar material prior to mitosis, thus increasing their microtubule nucleating capacity. Microtubules nucleated from the two centrosomes interdigitate in an antiparallel manner. These microtubules are moved apart by plus-end-directed motor proteins; a mechanism that indirectly pushes the two centrosomes to opposite poles of the cell and that supports the formation of a bipolar mitotic spindle. Spindle bipolarity is essential for the subsequent separation of the chromosomes into two daughter cells. Failures to separate chromosomes equally may result in aneuploid cells, which in turn are a hallmark of most human carcinomas. Because in many cancerous cells an elevated number of centrosomes has been detected, a causal link has been discussed between centrosome number and aneuploidy [1,2] (see other references therein). Clearly, supernumerary centrosomes are able to induce the formation of additional spindle poles during mitosis, thus segregating chromosomes to the extra pole. Because cytokinesis can occur even in the presence of extra spindle poles, daughter cells are produced that are missing the full complement of chromosomes. These daughter cells may not be viable if essential genetic information is lost. However, when only single chromosomes are missing, the homologous chromosome of the other parent could compensate. Such loss of heterozygosity may become a problem, for example if the remaining chromosome carries mutations in tumor suppressor genes. Multipolar spindles may also lead to daughter cells with supernumerary chromosomes, because cleavage during cytokinesis might occur asymmetrically, uniting multiple poles into one daughter cell. Moreover, cells with multipolar spindles might suffer more frequently from tension defects at kinetochore fibers, or from mono-oriented chromosomes, leading to the activation of the mitotic checkpoint. If the checkpoint control cannot be satisfied, the cells risk to abort mitosis, producing a tetraploid cell.

## Mechanisms leading to centrosome amplification

Currently, multiple mechanisms have been discussed that can lead to centrosome amplification. A leading hypothesis proposes that additional rounds of centrosome duplication during one cell cycle produces supernumerary centrosomes. Normally, the centrosome number is closely controlled by the protease separase that regulates the disconnection, or 'disengagement', of the centriole pair during anaphase and thereby licences centrosome duplication during S-phase of the following cell cycle [3]. However, overduplication of centrosomes can occur during prolonged S-phases, when DNA replication is inhibited by hydroxyurea [4,5]. In recent years, mounting evidence indicated that centrosomes can undergo additional, irregular cycles of duplication even after S-phase, at the time of DNA repair. In this context, multiple reports have indicated that genotoxic stress can lead to centrosome amplification [6-8]. More specifically, uncontrolled centrosome duplication has been demonstrated to occur when DNA repair during G2 phase is impaired, due to Rad51 knockout [9]. Besides Rad51, mutations or deficiencies of other proteins of the DNA repair mechanism such as BRCA1 and BRCA2 have been linked to centrosome amplification [10-16]. Moreover, the centrosome protein centrin 2 has been found to associate with the xeroderma pigmentosum group C complex that is involved in DNA repair [17-19]. Centrosome amplification seems to be favoured in cells lacking p53, and requires the activity of cdk2 in complex with cyclin A or cyclin E [20-23]. These are the cyclins found essential for regular centrosome duplication [24-27].

In addition to overduplication of centrosomes, other centrosome-related mechanisms have been described to induce multipolar spindles, such as splitting of centriole pairs, or the formation of acentriolar microtubule organising centers due to the accumulation of pericentriolar material [2,28,29]. A completely different mechanism leading to centrosome amplification has been proposed by Meraldi et al. [30]. These authors have shown that failure of cytokinesis due to overexpression of the kinases Aurora A, Aurora B, or Plk1 leads to binucleate cells containing two centrosomes. After duplication of their centrosomes as well as their DNA in S-phase, these cells would enter the next mitosis not only with four centrosomes but also with an octaploid genome.

Overall, the published literature reports a correlation between centrosome amplification and cancer. However, it is unclear whether centrosome amplification is a cause or rather a consequence of tumorigenesis. Moreover, multipolar spindles in cells with multiple centrosomes are expected to lead to frequent chromosome loss and produce non-viable daughter cells. Therefore, a mechanism must exist that counteracts multipolarity and that allows cancer cells with multiple centrosomes to proliferate. Quintyne et al. [31] reported that the microtubule motor dynein supports clustering of multiple centrosomes into a bipolar spindle apparatus. Such a mechanism could help cancer cells to undergo mitosis and maintain a karyotype that is optimal for proliferation.

## Cell cycle arrest in the absence of a functional centrosome

Whereas the presence of centrosomes has been correlated with proliferation, the loss of centrosomes has been found to block the cell cycle. Most interestingly, the loss of centrosomes from human cells did not prevent spindle formation in mitosis [32]. Instead, cells from which the centrosome was removed either by microsurgery or by laser ablation arrested at the following G1-S transition in the cell cycle [33,34]. Similar effects were seen after inhibition or silencing of several centrosome-associated proteins, such as dynactin, PARP-3, centriolin, and AKAP450 [35-38]. It was unclear, however, why cells would arrest in G1 after inhibition or removal of the centrosome. The centrosome could either play an essential role at the transition to S-phase, or alternatively the absence of an intact centrosome could trigger the checkpoint control system. To answer this question, Srsen et al. [39] monitored the effects of RNA silencing of two centrosome proteins, PCM-1 and pericentrin. The work indicated that depletion of either of these centrosome proteins increased the levels of the checkpoint control protein p53 and consequently of the cdk-inhibitor p21. The activation of p53 was in turn mediated by p38/MAP kinase that is known to phosphorylate and stabilize p53 as a response to cellular stress. The loss of centrosome proteins might therefore constitute a form of stress that activates p53. Although the loss of a functional centrosome in this experiment did not arrest all cells instantly, as half of these cells still proceeded into S-phase within four days after centrosome protein depletion, it predisposed them to undergo premature senescence. Senescence is a cellular program that responds to various physiological stresses and that leads to permanent cell cycle arrest [40]. However, cells undergoing senescence can stay alive for extended periods of time, in contrast to apoptotic cells. Contrary to previous belief, the data of Srsen et al. [39], as well as a recent report by Uetake et al. (Uetake Y, Lončarek J, Nordberg J, English C, Khodjakov A, Sluder G: The centrosome in G1 progression: important, but not essential. Abstract 965, 46^th ^annual meeting of the American Society for Cell Biology, San Diego, December 9–13, 2006) indicate that cells without a functional centrosome may prevent cell cycle progress due to a general increase of stress, rather than due to a specific activation of the G1-S checkpoint [41]. Because various kinases and phosphatases, as well as cyclin E and p53, have been localized to the centrosome [42-44], it seems likely that centrosome defects may interfere with cellular signalling pathways and therefore trigger a stress response, although the exact molecular details remain to be explored.

## Differentiation and loss of centrosome function

A correlation between loss of centrosome function and exit from the cell cycle has also been seen in various differentiating cell types during vertebrate development.

For example, in several epithelial cell types of liver, kidney, and intestine, the centrosome ceases to act as the microtubule organizing center upon polarization. Instead, centrosome proteins are localized in the apical region of the cell, or in a ribbon-like zone along the plasma membrane near the apex, such as in mouse cochlear cells [45-47]. A different type of reorganisation is observed in myoblasts undergoing differentiation into multinucleate muscle fiber cells. In these cells, centrosome proteins relocalize from the pericentriolar material to the outer nuclear surface [48,49]. At the same time, myoblasts withdraw from the cell cycle and become postmitotic. Interestingly, the signalling pathway leading to the differentiation of myoblasts and several other cell types, such as adipocytes and intestinal epithelial cells, has been found to involve the activation of p38/MAP kinase [50-55]. This means that exit from the cell cycle during differentiation is triggered by the same pathway as cell cycle arrest after experimental removal of centrosome proteins [39]. However, it is unclear whether there is a causal relationship between centrosome disassembly and p38-dependent exit from the cell cycle in differentiating cells. In particular, the observation of morphologically intact centrosomes in terminally differentiated cells such as neurons argues against the need of centrosome disassembly for cell cycle exit. Instead, activation of p38/MAP kinase, and therefore triggering of the signalling cascade that is also used in p38-dependent stress response, might activate p53 and p21-dependent cell cycle arrest and at the same time alter centrosome protein assembly. Consistently, altered solubility and altered assembly of the centrosome protein pericentrin have been seen in cells in which the stress pathway was activated by heat shock [56,57]. Such a mechanism would make sense, because once differentiating cells have withdrawn from the cell cycle, the spindle-forming activity of the centrosome is no longer needed, and disassembly or relocalization of centrosome proteins may help the cell in modulating the microtubule cytoskeleton, to fulfil specialized, differentiation-specific functions.

## Conclusion

Supernumerary centrosomes have frequently been found in a variety of cancer cells. It remains unclear whether supernumerary centrosomes are the driving force in proliferation and tumorigenesis, or whether centrosome amplification is a consequence of cancer development. In contrast, functional inhibition or removal of centrosome proteins leads to cell cycle arrest under experimental conditions. Understanding the regulatory mechanisms that link centrosome assembly to the cell cycle should be of immense value for the development of new strategies in cancer therapy.

## Competing interests

The author(s) declare that they have no competing interests.

## Authors' contributions

Both authors contributed equally to the conception and writing of this article.
